# Genetic testing considerations for hereditary duodenopancreatic neuroendocrine tumors

**DOI:** 10.1111/jne.70305

**Published:** 2026-11-02

**Authors:** Théo Charnay, Camille Giannetti, Nicolas Sahakian, Magalie Haissaguerre, Sandrine Oziel‐Taieb, Veronique Mari, Olivier Gilly, Pauline Vital, Isabelle Raingeard, Bénédicte Découdier, Alexandre Astier, Thomas Cuny, Pauline Romanet, Anne Barlier

**Affiliations:** ^1^ Aix Marseille Univ, APHM INSERM, MMG, MarMaRa Institute, La Timone University Hospital, Laboratory of Molecular Biology GEnOPé, BIOGENOPOLE Marseille France; ^2^ APHM, La Timone University Hospital Laboratory of Molecular Biology GEnOPé Marseille France; ^3^ Aix Marseille Univ, APHM, Inserm, MMG, MarMaRa Institute, La Conception University Hospital Department of Endocrinology Marseille France; ^4^ Haut Leveque University Hospital Department of Endocrinology Bordeaux France; ^5^ Institut Paoli Calmettes Medical Oncology Department, IPCNET Center, ENETS Center of Excellence Marseille France; ^6^ Oncogenetique Department Institut Antoine Lacassagne Nice France; ^7^ Service des Maladies Métaboliques et Endocriniennes CHU Nîmes Nîmes France; ^8^ Endocrinology Department CHU de Limoges Limoges France; ^9^ CHRU de Montpellier, Service d'Endocrinologie Diabète, Maladies Métaboliques Montpellier France; ^10^ CHU of Reims, Department of Endocrinology Hôpital Robert Debré Reims France; ^11^ Aix Marseille Univ, APHM, CNRS, INP, CHU Timone Oncobiology Laboratory Marseille France; ^12^ Clinical Investigation Center U1409, APHM Marseille France

**Keywords:** duodenopancreatic and pancreatic neuroendocrine tumor, familial tumor predisposition, germline genetic testing, multiple endocrine neoplasia type 1, neurofibromatosis type 1

## Abstract

The incidence of duodenal and pancreatic neuroendocrine tumors (dpNETs) has increased substantially in recent years. These tumors may be due to syndromic hereditary predisposition syndromes: multiple endocrine neoplasia type 1 (MEN1) or 4 (MEN4), Von Hippel–Lindau disease (VHL), neurofibromatosis type 1 (NF1), or tuberous sclerosis complex (TSC). Because a dpNET may be the first MEN1 sign, recent guidelines recommend *MEN1* genetic testing in selected patients with an isolated dpNET. Whether similar genetic testing strategies should be extended to other hereditary syndromes remains unknown. We conducted a retrospective analysis of the genetic and clinical data of patients who underwent genetic testing for isolated or syndromic dpNETs. We assessed the presence of (likely) pathogenic variants ((L)PVs) in *MEN1*, *NF1*, *VHL*, *CDKN1B*, *TSC1*, and *TSC2*. 410 patients were included: 301 with isolated (=non syndromic) dpNETs and 109 with syndromic dpNETs. Among them, 38 carried a (L)PV, most frequently in *MEN1* (*n* = 27 patients), followed by *NF1* (*n* = 8) and *VHL* (*n* = 3). No *TSC1*, *TSC2*, or *CDKN1B* (L)PVs were found. Patients with syndromic dpNETs exhibited significantly higher positive rates (29.4%) compared to isolated dpNETs (<2%). We confirm that *MEN1* is the predominant gene underlying predisposition to dpNETs. The low diagnostic yield in isolated dpNETs supports a clinically stratified genetic testing strategy. Clinical evaluation should systematically assess for signs of MEN1, MEN4, VHL, NF1, or TSC to inform appropriate genetic testing and maximize diagnostic yield. Concerning MEN1, we propose limiting extended genetic testing in patients with isolated dpNETs to those with (i) gastrinoma, (ii) multiple dpNETs, or (iii) diagnosis before 40 years of age.

## 1 INTRODUCTION

Neuroendocrine tumors (NETs) are a heterogeneous group of neoplasms arising from neuroendocrine cells that can secrete hormones and can occur throughout the body. The incidence of gastroenteropancreatic NETs has risen significantly over the years, reaching an estimated 5 per 100,000 individuals.^1^ Duodenal and pancreatic neuroendocrine tumors (dpNETs) may be associated with a syndromic hereditary predisposition (Table 1), most commonly multiple endocrine neoplasia type 1^2^ (MEN1, Online Mendelian Inheritance in Man (OMIM) #613733), due to inactivating genetic variants of the tumor suppressor gene *MEN1*.^3^, ^4^ Other syndromes that predispose to dpNETs have been described, including Von Hippel Lindau (VHL) disease, due to a *VHL* inactivating variant^5^ (OMIM #608537); multiple endocrine neoplasia type 4 (MEN4), due to a *CDKN1B* inactivating variant^6^ (OMIM #600778); neurofibromatosis type 1 (NF1), due to an *NF1* inactivating variant^7^ (OMIM #613113); and tuberous sclerosis complex (TSC), due to an inactivating variant in *TSC1* or *TSC2* ^8^ (OMIM #605284 and #191092). Other germline alterations have been reported in the literature such as *MUTYH*, *BRCA2*, and *CHEK2*.^9^, ^10^, ^11^, ^12^, ^13^ However, their causality in dpNET tumorigenesis remains controversial, due to a lack of supportive histological evidence and the absence of other somatic alterations (such as loss of heterozygosity [LOH] in the tumor), whereas an incidental association cannot be excluded. Furthermore, in two studies where an increased frequency of *BRCA2* variants was reported in pancreatic NETs, the findings were primarily attributed to founder effects within specific cohorts rather than a generalized predisposition to neuroendocrine tumors.^11^, ^14^

**TABLE 1:** Features of the main syndromes related to dpNETs.

Pathology	MEN1	MEN4	VHL	NF1	TSC
Gene	*MEN1*	*CDKN1B*	*VHL*	*NF1*	*TSC1* and *TSC2*
Disease prevalence	3–10/100,000	<<1/100,000	1–9/100,000	33/100,000	5/100,000
dpNET frequency	50–70%	12%	12%	0–10%	0.25–4%
Main dpNET characteristics	Multiple non‐functional 50% Gastrinoma 40%	Multiple Zollinger–Ellison syndrome	Multiple pancreatic: head 66%, body and tail 34%	Ampulla of Vater tumors, somatostatinoma	Pancreatic (slightly aggressive): body and tail 64%, multiple 29%
Other syndromic lesions	Primary hyperparathyroidism, pituitary adenoma, thymic neuroendocrine tumor	Primary hyperparathyroidism, pituitary adenoma, other neuroendocrine tumor	Hemangioblastoma, pancreatic and kidney cyst, cell renal carcinoma pheochromocytoma	Neurofibromas (cutaneous and plexiform) café‐au‐lait spots, Lisch nodules, optic pathway gliomas, distinctive skeletal abnormalities pheochromocytoma	Epilepsy, subependymal nodule, tuberous sclerosis complex‐associated neuropsychiatric disorder, cardiac rhabdomyoma, asymptomatic pulmonary lymphangioleiomyomatosis, focal hyperpigmentation, angiofibroma, angiomyolipoma

Abbreviations: dpNETs, duodenopancreatic neuro endocrine tumors; MEN1, multiple endocrine neoplasia type 1; MEN4, multiple endocrine neoplasia type 4; NF1, neurofibromatosis type 1; TSC, tuberous sclerosis complex; VHL, Von Hippel–Lindau.

Tumor growth and metastatic risk vary widely depending on endocrine function, on the tumor size, and on tumor genotype; for example, MEN1‐related insulinomas are multiple at the time of diagnosis and are associated with a metastatic risk up to 50%, whereas TSC‐related pancreatic NETs seem to have a lower metastatic risk, reported at approximately 19% in a recent cohort (5/26 patients), although the rarity of the syndrome restricts precise quantification.^15^, ^16^, ^17^ Given that curative treatment primarily relies on surgery for syndromic or functioning dpNETs or pancreatic NETs superior to 2 cm, early detection of dpNETs in patients with a genetic predisposition significantly improves survival outcomes.^18^, ^19^

Because a dpNET may be the first manifestation of a hereditary syndrome, a critical clinical question is whether patients presenting with an apparently isolated dpNET harbor germline pathogenic variants associated with syndromic predisposition. This concept is well established in MEN1, in which gastrinomas account for more than 40% of dpNETs and approximately 20% of patients with gastrinomas have MEN1.^20^ Accordingly, this clinical scenario is addressed in the most three recent MEN1 guidelines, which recommend germline genetic testing in selected patients presenting with apparently isolated dpNETs.^19^, ^21^, ^22^ Indeed, according to the characteristics of MEN1‐dependant dpNETs, *MEN1* genetic testing is recommended: (i) in patients with multiple or familial dpNETs at any age, (ii) in patients with dpNETs associated with Zollinger–Ellison syndrome (ZES), and (iii) in young patients with isolated and sporadic tumors.^2^, ^20^, ^22^ Whether similar testing strategies should be applied to identify other hereditary syndromes remains unknown. The advent of massive parallel sequencing now enables broad and efficient genetic screening; however, the clinical utility of genetic screening and prevalence of (likely) pathogenic variants ((L)PVs) in specific genes remain to be clarified in large dpNET cohorts of apparently non‐syndromic cases, here referred to as “isolated” cases.

Our objective was to evaluate the diagnostic yield of analyzing the six genes associated with hereditary syndromes predisposing to dpNETs (*MEN1*, *VHL*, *NF1*, *CDKN1B*, *TSC1*, and *TSC2*) in patients with isolated or syndromic dpNETs to refine genetic testing strategies. We therefore retrospectively analyzed the genetic and clinical data of these patients, who underwent broad endocrine tumor gene panel sequencing as part of their routine clinical care.

## 2 METHODS

### 2.1 Patients

We retrospectively analyzed genetic and clinical data from patients diagnosed with dpNETs who underwent genetic testing at the GEnOPé Molecular Biology Laboratory, Assistance Publique–Hôpitaux de Marseille between 2018 and 2024. Only index cases were selected; all data from relatives examined during familial screening were excluded from this analysis. Genetic testing was performed in cases of a syndromic dpNET related to one of the five syndromes (Table 1) or in cases of an isolated dpNET, in accordance with current French recommendations.^2^ These recommendations include testing for: (i) multiple or familial dpNETs, (ii) dpNETs associated with Zollinger–Ellison syndrome (ZES), and (iii) patients with isolated and sporadic tumors diagnosed before the age of 50. Notably, 93 patients with an isolated dpNET diagnosed after 50 years of age underwent testing outside these guidelines. Clinical data were recorded at the time of genetic testing and excluded any lesions identified during subsequent investigations prompted by positive genetic results.

In the present study, patients were considered to have isolated dpNETs (i.e., non‐syndromic) if they presented with dpNETs only, whether single, multiple, or metastatic, and as syndromic when their disease was associated with extra‐pancreatic or duodenal manifestations characteristic of one of the five hereditary syndromes predisposing to dpNETs: MEN1, MEN4, VHL, TSC, or NF1.

Cases were classified as familial if at least one relative had either (i) a dpNET or (ii) a syndrome‐associated lesion; otherwise, they were deemed sporadic.

All patients provided written informed consent for germline genetic analysis. Clinical data were collected from the clinician's genetic test request form. This study was approved by the APHM Centre Research Ethics Board (PADS24‐321).

### 2.2 Genetic testing

Genomic DNA was extracted from EDTA blood samples on a QiaSymphony DS instrument (Qiagen). Next generation sequencing (NGS) library preparation targeted all exons and 20‐bp flanking introns of *MEN1* NM_130799.3, *VHL* NM_000551.3, *CDKN1B* NM_004064.4, *NF1* NM_001042492.2, *TSC1* NM_000368.4, and *TSC2* NM_000548.4 using a QIAseq Targeted DNA Custom Panel (Qiagen) and were sequenced on a NextSeq1000 sequencer (Illumina). Sequence alignment to the human genome (GRCH37/hg19), variant calling of variant with allelic frequency (VAF) superior to 5%, and annotation were performed using the CLC Genomic Workbench software (Qiagen). All regions of interest were sequenced with a depth of coverage superior to 30×.

### 2.3 Mosaicism detection

For patients with highly specific phenotypes (meaning at least two lesions of the syndrome), low‐level mosaicism was sought in blood DNA by screening variants with a VAF between 1% and 5%^23^ through deep sequencing. Mosaicism was considered negative if no variant was detected at a sequencing depth >300×. Variants were confirmed by searching for the variant either in the syndromic lesions when these were available or in a second blood sample from the patient, as previously described.^24^

### 2.4 Classification of variants

Each variant was classified according to ACMG‐AMP and ClinGen guidelines into one of five classes of pathogenicity^25^, ^26^: class 1: benign variant (BV); class 2: likely benign variant (LBV); class 3: variant of uncertain significance (VUS); class 4: likely pathogenic variant (LPV); or class 5: pathogenic variant (PV). Computational and population data were aggregated using Alamut Visual software (Interactive Biosoftware, Rouen, France) and MobiDetails.^27^ UMD‐Predictor,^28^ SIFT, PolyPhen‐2, Revel, HSF (https://hsf.genomnis.com/), and spliceAI were used for in silico predictions. The population data were collected from the normal population database (gnomAD database v4.1, last accessed 11/08/2025) and from inherited disease databases (ClinVar, LOVD, UMD‐MEN1 database, and HGMD). BVs and LBVs were not reported in this study.

### 2.5 Statistical analyses

Statistical analyses were performed using BiostaTGV software (Sentiweb, INSERM, France). Categorical variables were compared using the Chi‐square test. When the expected number of cases in any cell was less than 5, Yates' continuity correction was applied to ensure the validity of the results. For the comparison of age at time of tumor detection between different patient subgroups, the nonparametric Mann–Whitney *U* test was employed. Statistical significance was defined as a *p*‐value <.05.

## 3 RESULTS

### 3.1 Characterization of the dpNET cohort

We collected data from 410 index cases presenting at least one dpNET, 73.4% of these having an isolated dpNET and 26.6% a syndromic dpNET (Table 2). A slight female predominance was observed. The age of patients at dpNET diagnosis ranged from 15 to 87 years of age, with a median of 49 years. Cases were classified as familial if at least one relative had either (i) a dpNET (*n* = 7) or (ii) a syndrome‐associated lesion (*n* = 19); otherwise, they were deemed sporadic. Familial cases represented 6.3% of the whole cohort, 17.4% of the 109 syndromic patients (including some overlapping syndromes), and 2.3% of the 301 patients with isolated dpNETs (Table 2).

**TABLE 2:** Clinical characteristics of patients at the time of genetic testing.

		Total *n* = 410	Syndromic *n* = 109 (26.6%)	Isolated *n* = 301 (73.4%)
Male/female (%)		176/234 (42.9/57.1%)	45/64 (41.3/58.7%)	131/170 (43.5/56.5%)
Familial (%)		26/410 (6.3%)	19/109 (17.4%)	7/301 (2.3%)
Age median (years min–max)		49 (15–87)	51 (16–87)	47 (15–81)
Location	Pancreatic	319 (77.8%)	73 (67%)	246 (81.7%)
	Duodenal	16 (3.9%)	7 (6.4%)	9 (3%)
	Duodenal or pancreatic^a^	75 (18.2%)	29 (26.6%)	46 (15.3%)
dpNET	Single dpNET	334 (81.5%)	83 (76.1%)	251 (83.4%)
	Multiple dpNETs	57 (13.9%)	23 (21.1%)	34 (11.3%)
	Metastatic dpNETs	19 (4.6%)	3 (2.8%)	16 (5.3%)
Endocrine function	Insulinoma	66 (16.1%)	13 (11.9%)	53 (17.6%)
	Zollinger–Ellison syndrome/gastrinoma	31 (7.6%)	13 (11.9%)	18 (6%)
	Glucagonoma	6 (1.5%)	1 (0.9%)	5 (1.7%)
	VIPome	5 (1.2%)	1 (0.9%)	4 (1.3%)
	Non‐functional	302 (73.6%)	81 (74.3%)	221 (73.4%)

*Note*: Sex ratio is presented as number of individuals (male/female), followed by the corresponding percentages in overall population. Percentages are calculated in the whole population (left column), in the syndromic population (middle column), or in the isolated dpNET population (right column).

Abbreviation: dpNETs, duodenopancreatic neuro endocrine tumors.

^a^ The duodenal or pancreatic location was not specified by the clinician.

In terms of tumor burden, most patients presented with a single dpNET (81.5%), while 13.9% had multiple tumors, and 4.6% presented with metastatic disease at diagnosis. Regarding endocrine function, 73.6% of the tumors were non‐functional and 26.4% were functional. Within the functional group (*n* = 108), insulinomas were the most frequent subtype (61%), followed by gastrinomas (29%; Table 2).

### 3.2 Germline genetic description of the whole cohort

A (L)PV was detected in 9.2% of patients (*n* = 38) of the whole cohort. In the majority of cases, it was a private (L)PV (Table S1, Supporting Information). The distribution of (L)PVs in these 38 patients was as follows: 27 patients harbored a *MEN1* (L)PV (71% of the positive patients), eight harbored an *NF1* (L)PV (21%), and three harbored a *VHL* (L)PV (7.8%). No (L)PVs were identified in *CDKN1B*, *TSC1*, or *TSC2*.

Notably, mosaicism was detected in four patients (10% of the positive cases): three in syndromic *MEN1* (with blood VAF ranging from 3.6% to 19%), all of whom were previously reported^23^ and one in syndromic *VHL* (VAF of 12%; Table S1).

Additionally, 22 VUS were identified in 20 patients (Table S1). These were distributed across *MEN1* (*n* = 5), *NF1* (*n* = 4), *TSC1* (*n* = 5), and *TSC2* (*n* = 8).

### 3.3 Characteristics of patients with a (L)PV

The percentage of (L)PV‐positive patients was higher in patients harboring syndromic dpNETs (29.4%) than those with isolated tumors (1.9%, *p* < .0001; Table 3). The significantly higher detection rate in the syndromic group was consistently observed regardless of the number of dpNETs (single vs. multiple). Within the syndromic group, the (L)PV detection rate was significantly higher in familial cases (68.4%) than in sporadic cases (21%, *p* < .001). Conversely, no (L)PVs were identified in the familial isolated dpNET group (Table 3).

**TABLE 3:** Clinical characteristics at the time of genetic testing of patients with a (likely) pathogenic variant.

Patients with (L)PV		Total *n* = 38 (38/410) 9.3%	Syndromic *n* = 32 (32/109) 29.4%	Isolated *n* = 6 (6/301) 1.9%	*p* < .001
Male/female total		18/20	15/17	3/3	NS
*MEN1*		13/14	10/12	3/2	
*NF1*		3/5	3/4	0/1	
*VHL*		2/1	2/1	0/0	
Median age (years min–max)		43.5 (19–69)	44 (20–69)	37 (19–69)	NS
*MEN1*		43 (19–69)	46 (20–69)	36 (19–60)	
*NF1*		52 (33–69)	51 (33–63)	69	
*VHL*		22 (20–27)	22 (20–27)	No patient	
Sporadic [MEN1/NF1/VHL]		25/384 (6.5%) [19M/4N/2V]	19/90 (21%) [14M/3N/2V]	6/294 (2%) [5M/1N/0V]	*p* < .001
Familial [MEN1/NF1/VHL]		13/26 (50%) [8M/4N/1V]	13/19 (68.4%) [8M/4N/1V]	0/7 [0M/0N/0V]	*p* = .008
Localization	Pancreatic	25/319 (7.8%)	19/73 (26%)	6/246 (2.4%)	*p* < .001
	Duodenal	3/16 (18.8%)	3/7 (42.9%)	0/9	NS
	Duodenopancreatic^a^	10/75 (13.3%)	10/29 (34.5%)	0/46	*p* < .001
dpNET	Single dpNET [MEN1/NF1/VHL]	25/334 (7.5%) [18M/6N/1V]	20/83 (24.1%) [14M/5N/1V]	5/251 (2.3%) [4M/1N/0V]	*p* < .001
	Multiple dpNETs [MEN1/NF1/VHL]	13/57 (22.8%) [9M/2N/2V]	12/23 (52.2%) [8M/2N/2V]	1/34 (2.9%) [1M/0N/0V]	*p* = .03
	Metastatic dpNETs [MEN1/NF1/VHL]	0/19 [0M/0N/0V]	0/3 [0M/0N/0V]	0/16 [0M/0N/0V]	NS
Endocrine function	Insulinoma [MEN1/NF1/VHL]	3/66 (4.5%)^1^ ^b^ [3M/0N/0V]	3/13 (23.1%) [3M/0N/0V]	0/53^1^ ^b^ [0M/0N/0V]	*p* = .004
	ZE syndrome/gastrinoma [MEN1/NF1/VHL]	6/31 (19.4%)^4^ ^b^ [2M/4N/0V]	5/13 (38.4%)^2^ ^b^ [2M/3N/0V]	1/18 (5.6%)^2^ ^b^ [0M/1N/0V]	NS
	Glucagonoma [MEN1/NF1/VHL]	0/6 [0M/0N/0V]	0/1 [0M/0N/0V]	0/5 [0M/0N/0V]	NS
	VIPome [MEN1/NF1/VHL]	0/5 [0M/0N/0V]	0/1 [0M/0N/0V]	0/4 [0M/0N/0V]	NS
	Non‐functional [MEN1/NF1/VHL]	29/302 (9.6%)^14^ ^b^ [22M/4N/3V]	24/81 (29.6%)^1^ ^b^ [17M/4N/3V]	5/221 (2.3%)^13^ ^b^ [5M/0N/0V]	*p* < .001
Patients with *MEN1* (L)PV [pancreatic/duodenal/duodenopancreatic^a^]		27/410 (6.5%) [20/1/6]	22/109 (20.2%) [15/1/6]	5/301 (1.6%) [5/0/0]	*p* < .001
Patients with *VHL* (L)PV [pancreatic/duodenal/duodenopancreatic^a^]		3/410 (0.7%) [3/0/0]	3/109 (2.7%) [3/0/0]	0/301 [0/0/0]	*p* = .026
Patients with *NF1* (L)PV [pancreatic/duodenal/duodenopancreatic^a^]		8/410 (2%) [2/2/4]	7/109 (6.4%) [1/2/4]	1/301 (0.3%) [1/0/0]	*p* < .001

*Note*: The results are expressed as the number of patients with a (likely) pathogenic variant/number of patients in each category with the corresponding percentage. *p*‐value calculated between isolated and syndromic groups (not between *MEN1/NF1/VHL* subgroups).

Abbreviations: dpNETs, duodenopancreatic neuro endocrine tumors; (L)PV, (likely)pathogenic variant; M, *MEN1*; N, *NF1*; NS, not significant; V, *VHL*; ZE, Zollinger–Ellison.

^a^ The duodenal or pancreatic location was not specified.

^b^ Including patients with metastasis.

Patients with a (L)PV were significantly younger at diagnosis of dpNET (median: 43.5 years, range 19–69) as compared to patients without any (L)PV (50 years, range 15–87, *p* = .0034). Moreover, patients (*n* = 3) carrying a *VHL* (L)PV exhibited an earlier onset of dpNETs (median age: 22 years), compared to patients with a *MEN1* (L)PV (*n* = 22, median age: 43 years, *p* = .024) or patients with an *NF1* (L)PV (*n* = 8, median age: 52 years, *p* = .012; Table 3).

Among the single isolated sporadic dpNET group of patients (*n* = 244), in most cases, the age at dpNET diagnosis was over 40 years (*n* = 178, including 76 patients aged between 40 and 50 years), with only 66 patients diagnosed at an age younger than 40 years. The (L)PV prevalence reached 6.1% (4/66) in this group of young patients, whereas no (L)PVs were identified in those with dpNETs diagnosed at age 40 or older (Table 4).

**TABLE 4:** Positive rate according to the age of the syndromic or isolated (=non syndromic) context, the age of duodenopancreatic neuroendocrine tumor occurrence, the familial context, and the tumor phenotype.

	Syndromic		Isolated	
	Familial	Sporadic	Familial	Sporadic
Under 40 years				
Single	100% (5/5)^2^ ^a^	30% (3/10)	0% (0/2)	6.1% (4/66)^2^ ^a^
Multiple	75% (3/4)	50% (3/6)	0% (0/0)	0% (0/4)
Metastatic	0% (0/0)	0% (0/0)	0% (0/0)	0% (0/6)^2^ ^a^
Between 40 and 50 years				
Single	50% (1/2)	14.3% (2/14)^1^ ^a^	0% (0/0)	0% (0/76)^4^ ^a^
Multiple	0% (0/1)	100% (1/1)	0% (0/0)	0% (0/8)
Metastatic	0% (0/1)	0% (0/0)	0% (0/0)	0% (0/2)
Older than 50 years				
Single	75% (3/4)^1^ ^a^	12.5% (6/48)^7^ ^a^	0% (0/5)	1% (1/102)^9^ ^a^
Multiple	100% (1/1)	40% (4/10)	0% (0/0)	4.5% (1/22)^1^ ^a^
Metastatic	0% (0/1)^1^ ^a^	0% (0/1)^1^ ^a^	0% (0/0)	0% (0/8)

*Note:* Cells shaded dark grey indicate a high probability of genetic diagnosis (>20%); light grey, a moderate probability (2‐19%); and unshaded cells, a low probability (<2%).

^a^ Including patients with Zollinger–Ellison syndrome or gastrinoma.

In the group with multiple isolated sporadic dpNETs, regardless of endocrine phenotype (*n* = 34, with four patients diagnosed before age 40, and eight between 40 and 50 years of age; Table 4), the (L)PV rate was 3% (1/34). Notably, this single positive case involved a patient whose tumors were first diagnosed at age 60.

Concerning the endocrine phenotype, the percentage of patients with a (L)PV was higher in the group of patients with gastrinomas compared to other functional dpNETs (19.4% (6/31) vs. 4.6% (3/77), *p* = .024; Table 3). Lastly, no (L)PV was identified in the group with metastatic dpNETs (0/19).

### 3.4 Characterization of patients with a (L)PV according to each gene

Overall, *MEN1* (L)PVs were identified in 27 patients across the whole cohort (6.5%; Table 3). Within the MEN1 syndromic group (*n* = 90), the *MEN1* (L)PV detection rate was 24%, including three syndromic patients with *MEN1* constitutional mosaicism (Table 5). The prevalence of germline variants increased with the number of MEN1‐related lesions: 20% (13/66) in patients with two MEN1‐related lesions and 37% (9/24) in those with three or more (Table 5). This correlation was even more pronounced in familial cases, where the mutation rate reached 50% (5/10) in patients with two lesions and 75% (3/4) in patients with three or more lesions. In terms of the sequence of diagnosis, the dpNET was the presenting feature in 18.5% of genetically confirmed MEN1 cases (5/27). In the group of patients with isolated dpNETs (*n* = 301), five (1.6%) were tested positive for *MEN1*; four patients were diagnosed before the age of 40, while the fifth patient presented with multiple dpNETs at age 60. Regarding the endocrine function of dpNETs of the whole cohort (Table 3), a *MEN1* (L)PV was found in two of the 31 (6%) patients bearing a gastrinoma, in three out of the 66 patients (4.5%) bearing an insulinoma, and in 22 out of the 302 patients (7.3%) bearing nonfunctional dpNETs.

**TABLE 5:** Patients with *MEN1*, *VHL*, or *NF1 positive* genetic testing according to their clinical presentation at the time of genetic testing.

	All	Sporadic	Familial
*MEN1*‐positive patients among patients with other MEN1‐related lesion(s)^a^, ^b^	22/90 (24%)	14/76 (18%)	8/14 (57%)
dpNET + PHPT	13/55 (23%)	8/46 (17%)	5/9 (55%)
dpNET + pituitary adenoma	0/11 (0%)	0/10 (0%)	0/1 (0%)
dpNET + PHPT + pituitary adenoma or other NET	9/24 (37%)	6/16 (37%)	3/4 (75%)
*MEN1*‐positive patients among patients with isolated dpNETs	5/301 (1.6%)	5/294 (1.7%)	0/7
*VHL*‐positive patients among patients with other *VHL*‐related lesion(s)^a^	3/19 (16%)	2/17 (12%)	1/2 (50%)
Pheochromocytoma/paraganglioma	0/8	0/8	0/0
Renal cyst or carcinoma	1/6 (16%)	1/6 (16%)	0/0
Hemangioblastoma	2/2 (100%)	1/1 (100%)	1/1 (100%)
*VHL*‐positive patients among patients with isolated dpNETs	0/301	0/294	0/7
*NF1*‐positive patients among patients with other *NF1*‐related lesion(s)^b^	7/14 (50%)	3/10 (30%)	4/4 (100%)
NF1 cutaneous lesions	6/6 (100%)	2/2 (100%)	4/4 (100%)
Pheochromocytoma/paraganglioma	1/8 (12.5%)	1/8 (12.5%)	0/0
*NF1*‐positive patients among patients with isolated dpNETs	1/301 (0.3%)	1/294 (0.3%)	0/7

Abbreviations: dpNETs, duodenopancreatic neuro endocrine tumors; (L)PV, (likely) pathogenic variant; NET, neuro endocrine tumors; PHPT, primary hyperparathyroidism.

^a^ Four patients presented with both MEN1 and VHL phenotypes, i.e., a dpNET and a PHPT occurring in association with a pheochromocytoma, paraganglioma, or renal cell carcinoma. (L)PV were excluded in all candidate genes associated with PPGL or RCC.

^b^ One patient presented with a dpNET, PHPT, and a clinical diagnosis of NF1.

*VHL* (L)PVs were identified in three patients across the whole cohort (0.7%; Table 3). All *VHL* (L)PVs were identified in patients from the VHL syndromic group (3/19, 16%), whereas no (L)PVs were found in patients with isolated dpNETs (Table 5). Two patients harbored both a dpNET and hemangioblastoma, and both had a *VHL* (L)PV. All VHL‐related dpNETs were nonfunctional tumors.

*NF1* (L)PVs were identified in eight patients across the whole cohort (2%; Table 3). Unexpectedly, one of them presented with an isolated dpNET as the presenting feature that led to NF1 diagnosis through genetic testing, whereas the remaining seven patients had clinical criteria of NF1 syndrome. Notably, gastrinomas accounted for 50% (*n* = 4) of the dpNETs in the *NF1*‐positive patients.

## 4 DISCUSSION

We report here a large series of genetic testing in patients with dpNETs including 301 patients with isolated dpNETs and 109 syndromic dpNETs.

The presence of syndromic manifestations, where the prevalence of (L)PVs reaches 29.4%, should guide genetic testing. The association of a dpNET with a pheochromocytoma/paraganglioma (*n* = 8 in our series) warrants analysis of established predisposition genes^29^, ^30^ including not only *VHL* but also *NF1*. Notably, one documented case has reported a syndromic dpNET associating oligodendroglioma, glomus tumors, and a *SDHD* (L)PV,^31^ suggesting that *SDHx* genes remain to be assessed in these rare associated lesions.

Our findings reaffirm the predominant role of *MEN1* as the leading genetic driver of hereditary dpNETs. Among the six patients with an isolated sporadic dpNET carrying a germline (L)PV, five harbored a *MEN1* variant, confirming that a dpNET can be the heralding manifestation of MEN1.

While established clinical practice guidelines and the existing literature suggest a higher penetrance of dpNETs in VHL disease (9%–17%) compared to NF1 (2.8%–10%),^4^, ^5^, ^7^, ^32^ we observed in our series a higher number of (L)PVs in *NF1* than *VHL*. This discrepancy likely stems from the tenfold higher prevalence of NF1 in the general population (1/3000) compared to VHL (1/30,000). Consequently, even if the risk of developing a dpNET is lower for an individual patient with NF1, the absolute number of NF1‐related dpNETs in the population may exceed those related to VHL.

In 2001, Tanaka et al. showed a strong association between NF1 and the occurrence of somatostatinomas, particularly those located in the ampulla of Vater.^33^ Interestingly, although our cohort included five non‐functional somatostatinomas (one ampullary), none of these patients carried a *NF1* (L)PV. Instead, gastrinomas were identified in 50% (4/8) of *NF1‐positive* patients, suggesting that the phenotypic spectrum of NF1‐associated dpNETs may be broader than previously reported. The direct involvement of *NF1* in neuroendocrine tumorigenesis is supported in the literature by genomic analysis showing a LOH at the *NF1* locus within the tumor.^34^ An unexpected finding was the identification of a patient with NF1 through genetic testing despite the absence of clinical features suggestive of the syndrome. This patient presented with an apparently isolated dpNET, and the discovery of the germline (L)PV prompted a retrospective clinical re‐evaluation which identified subtle café‐au‐lait spots. This case illustrates that a dpNET can indeed be the index event of NF1 syndrome, highlighting the clinical utility of including *NF1* in genetic screening panels, and the important need for increased clinical awareness regarding NF1 lesions.

In line with previous studies, our findings support that a dpNET is not the heralding manifestation of VHL,^35^ while no *VHL* (L)PVs were identified in patients with isolated dpNETs.

Based on our findings, we refined the indications for germline genetic testing according to clinical presentation, as illustrated in Figure 1. In this proposition, metastatic status is not included. Metastatic dpNETs have been reported to be less frequently diagnosed in patients with genetic syndromes compared to isolated cases.^36^, ^37^, ^38^ In our study, metastatic disease was not found associated with a germline (L)PV. Consequently, in patients with metastatic tumors, somatic genetic profiling may represent the preferred first‐line approach, as it can both inform clinical management and, when appropriate, prompt targeted germline testing.

**FIGURE 1:**
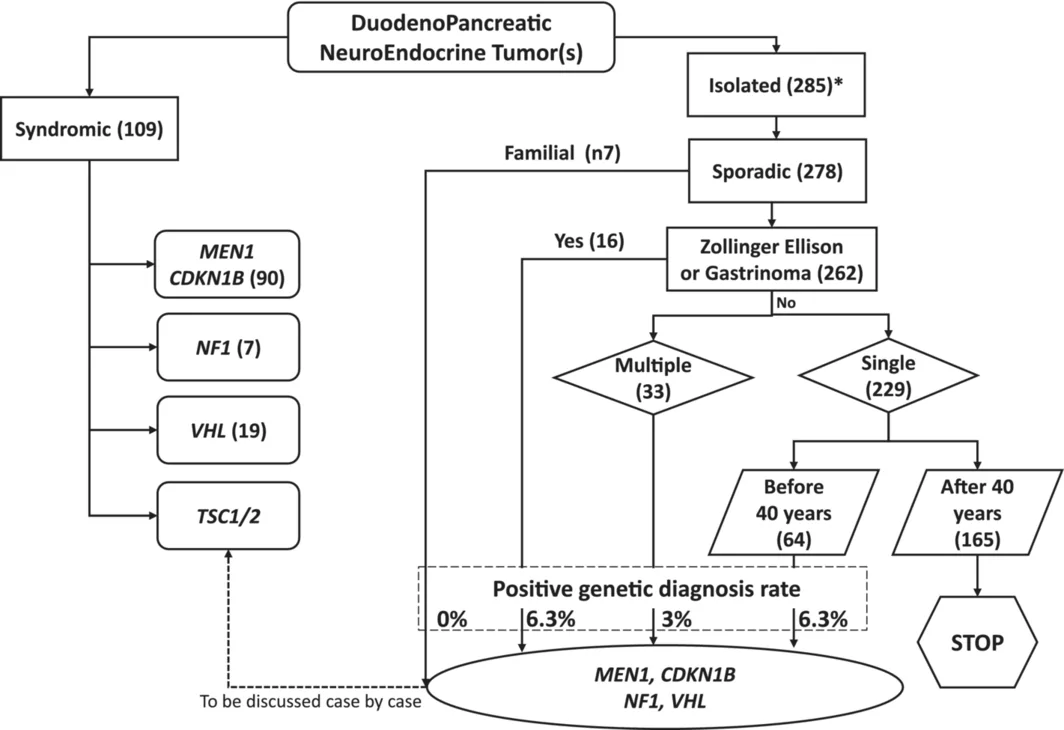
Refined indications for germline genetic testing derived from literature and the study findings. Numbers in parentheses represent the number of patients in each group from the present study. *Metastatic dpNETs were excluded.

Familial isolated dpNETs are rare, accounting for only 2.3% (7/301) of patients with isolated dpNETs in our series. No (L)PV was found in these seven patients. No conclusion on the utility of genetic testing in this sub‐group could be addressed based on this study. As a precaution, we recommend that these patients undergo genetic testing until additional data on non‐syndromic familial dpNETs (which appear to be an exceptional condition) are published.

While approximately 40% of MEN1 patients develop gastrinomas,^20^ our findings showed that among 16 patients with apparently non‐syndromic, sporadic ZES/gastrinoma, only one carried a (L)PV (6.3%), surprisingly in *NF1* and not in *MEN1*. Indeed, ZES is rarely, if ever, the inaugural manifestation of MEN1 disease; gastrinomas in MEN1 context are typically diagnosed in patients with pre‐existing primary hyperparathyroidism.^39^ Nevertheless, the high proportion of genetic syndromes identified in the ZES/gastrinoma group (19.4%; Table 3) supports systematic genetic screening regardless of the initial clinical presentation, as illustrated in the decision tree proposed in this study (Figure 1).

In contrast to the situation where multiple tumors lead to genetic testing regardless of the phenotype and the age, in patients with isolated, sporadic, single non‐gastrinoma dpNETs, an age threshold for recommending genetic testing should be established. Our findings indicate that the age threshold should be 40 rather than 50 years (Figure 1), as suggested by recent MEN1 recommendations.^22^, ^40^ The previous threshold of 50 years, initially recommended in France, was based on the peak incidence of dpNETs occurring after 50–55 years of age.^41^ The use of a 40‐year age limit in our series increased the rate of diagnosis from 2.9% (4/136) to 6.3% (4/64), without a missed diagnosis (Figure 1).

No (L)PVs for *TSC1* or *TSC2* were detected in our cohort, likely due to the low incidence of TSC. These data, along with the presence of a substantial burden of VUS (accounting for 13 *TSC1/2* VUS among a total of 22 VUS, due to the highly polymorphic nature of these genes), do not support the inclusion of these genes in routine screening panels. The limitation is not primarily a sequencing issue but rather the time‐consuming nature of allelic variant interpretation, which remains a significant challenge. In TSC patients, the prevalence of dpNETs is estimated around 0.24% (5 cases among 2093 TSC patients) in a European cohort,^8^ around 1.1% in a recent study from the United Kingdom,^17^ and around 1.5% to 2% across two different American cohorts.^42^, ^43^ LOH *i*n *TSC1* and *TSC2* has been well documented in several studies, supporting their role in dpNET tumorigenesis.^44^, ^45^ Consequently, screening of *TSC1* and *TSC2* remains clinically relevant for dpNETs with TSC‐related lesions.

MEN4 is a very rare disease. The prevalence of dpNETs was estimated at 12% (four patients with dpNETs among 33 CDKN1B‐mutation–positive patients). These 33 patients comprised 29 from a worldwide literature review, after careful variant reclassification according to ACMG criteria, and four index cases identified among 5600 genetic test records from the French oncogenetic network TENGEN.^6^ According to this last study, MEN4‐associated dpNETs occur less frequently and at a later age than those related to MEN1. Given the phenotypic overlap with MEN1 and the small size of the *CDKN1B* gene (3 exons, 597 coding bases), we recommend including *CDKN1B* in second line syndromic dpNET panels. However, further studies are required to confirm this, as cost‐effectiveness considerations may influence its systematic analysis.

To finish, the low detection rate in the isolated group, together with the absence of positive patients in the familial isolated group, raises the question of additional candidate genes specific to dpNETs. Although mutations in genes such as *GCGR* (associated with Mahvash disease),^46^, ^47^ and *MAFA* (linked to familial insulinomatosis)^48^, ^49^ have been documented, these conditions remain exceptionally rare and present with distinct phenotypes. Given the very low prevalence of these specific presentations, routine screening for these genes is not justified and should be strictly reserved for patients with a highly suggestive clinical or family history.

Single‐gene testing has largely been replaced by broad multigene panel testing. However, one major challenge of this approach is the identification of incidental findings and VUS. In constitutional genetics, it is likely preferable to restrict NGS data analysis to the specific clinical setting. This targeted approach not only avoids incidental findings, which can cause anxiety for patients, but also reduces the risk of detecting VUS that do not contribute to clinical decision‐making. Furthermore, limited gene panels enable deep sequencing, a critical requirement for detecting genetic mosaicism, as observed in four cases in our study.

## 5 CONCLUSION

We confirm that *MEN1* is the predominant gene underlying predisposition to dpNETs. When evaluating a patient with a dpNET, our data highlight the importance of carefully examining patients for clinical features suggestive not only of classical MEN1/MEN4 or VHL but also of NF1 and TSC to guide appropriate genetic testing and maximize diagnostic yield. Although syndromic dpNET presentation can guide genetic testing, the challenge persists in cases of isolated dpNETs, which have a low diagnostic yield.

To address this, we proposed a clinically based stratified screening strategy to optimize mutation detection rates while minimizing unnecessary testing and VUS detection. As for *MEN1*, we propose limiting extended genetic testing in patients with isolated dpNETs to those with (i) gastrinoma, (ii) multiple dpNETs, or (iii) a diagnosis before 40 years of age.

## AUTHOR CONTRIBUTIONS

**Théo Charnay:** Conceptualization; software; formal analysis; investigation; writing – original draft; writing – review and editing. **Camille Giannetti:** Data curation; formal analysis; investigation; writing – original draft. **Nicolas Sahakian:** Investigation; writing – review and editing. **Magalie Haissaguerre:** Investigation; writing – review and editing. **Sandrine Oziel‐Taieb:** Investigation; writing – review and editing. **Veronique Mari:** Investigation; writing – review and editing. **Olivier Gilly:** Investigation; writing – review and editing. **Pauline Vital:** Investigation; writing – review and editing. **Isabelle Raingeard:** Investigation; writing – review and editing. **Bénédicte Découdier:** Investigation; writing – review and editing. **Alexandre Astier:** Investigation; writing – review and editing. **Thomas Cuny:** Investigation; writing – review and editing. **Pauline Romanet:** Conceptualization; project administration; investigation; writing – original draft; writing – review and editing. **Anne Barlier:** Conceptualization; investigation; writing – original draft; writing – review and editing.

## FUNDING INFORMATION

This work received support from the French government under the France 2030 investment plan, as part of the Initiative d'Excellence d'Aix‐Marseille Université‐A*MIDEX (AMX‐19‐IET‐007) through the Marseille Maladies Rares Institute (MarMaRa).

## CONFLICT OF INTEREST STATEMENT

The authors declare no conflicts of interest.

## ETHICS STATEMENT

All patients provided written informed consent for germline genetic analysis. Clinical data were collected from the clinician's genetic test request form. This study was approved by the APHM Centre Research Ethics Board (PADS24‐321).

## Supporting information


**Table S1.** Clinical data and ACMG classification of 34 (likely) pathogenic variants and 22 variants of unknown significance.

## Data Availability

The data that support the findings of this study are available on request from the corresponding author. The data are not publicly available due to privacy or ethical restrictions.
